# Supplementary material for: Chilean Salmon Sushi: Genetics Reveals Product Mislabeling and a Lack of Reliable Information at the Point of Sale
Source: Foods. 2020 Nov 19;9(11):1699. doi: 10.3390/foods9111699 (PMC7699462; doi:10.3390/foods9111699)
Supplement: Supplementary file 1 [file foods-09-01699-s001.zip › Table S2.docx]

Table S2. Localities, sushi places sampled along Chile and molecular diagnosis.

| **Locality** | **Sushi place^1^** | **Declared in the menu^2^** | **Declared by the salesperson^3^** | **RFLP^4^** |
| --- | --- | --- | --- | --- |
| Iquique | Restaurant 1 | Salmon | NS | Atlantic salmon |
|  | Restaurant 2 | Salmon | NS | Atlantic salmon |
|  | Restaurant 3 | Salmon | NS | Atlantic salmon |
|  | Restaurant 4 | Salmon | NS | Atlantic salmon |
|  | Restaurant 5 | Salmon | NS | Rainbow trout |
|  | Restaurant 6 | Salmon | NS | Atlantic salmon |
|  | Restaurant 7 | Salmon | NS | Atlantic salmon |
|  | Restaurant 8 | Salmon | NS | Atlantic salmon |
|  | Restaurant 9 | Salmon | NS | Atlantic salmon |
|  | Restaurant 10 | Salmon | NS | Atlantic salmon |
|  | Restaurant 11 | Salmon | NS | Atlantic salmon |
| Antofagasta | Restaurant 1 | Salmon | NS | Atlantic salmon |
|  | Restaurant 2 | Salmon | NS | Atlantic salmon |
|  | Restaurant 3 | Salmon | Atlantic salmon | Atlantic salmon |
|  | Restaurant 4 | Salmon | NS | Atlantic salmon |
|  | Restaurant 5 | Salmon | NS | Atlantic salmon |
|  | Restaurant 6 | Salmon | NS | Atlantic salmon |
|  | Restaurant 7 | Salmon | NS | Atlantic salmon |
| Viña del Mar | Restaurant 1 | Salmon | NS | Atlantic salmon |
|  | Restaurant 2 | Salmon | NS | Rainbow trout |
|  | Restaurant 3 | Salmon | Atlantic salmon | Rainbow trout |
|  | Restaurant 4 | Salmon | Atlantic salmon | Atlantic salmon |
|  | Restaurant 5 | Salmon | NS | Atlantic salmon |
|  | Restaurant 6 | Salmon | NS | Rainbow trout |
|  | Restaurant 7 | Salmon | NS | Atlantic salmon |
|  | Restaurant 8 | Salmon | NS | Rainbow trout |
|  | Restaurant 9 | Salmon | NS | Rainbow trout |
|  | Restaurant 10 | Salmon | NS | Atlantic salmon |
| Valparaíso | Restaurant 1 | Salmon | NS | Rainbow trout |
|  | Restaurant 2 | Salmon | NS | Rainbow trout |
|  | Restaurant 3 | Salmon | Atlantic salmon | Atlantic salmon |
|  | Restaurant 4 | Salmon | Atlantic salmon | Rainbow trout |
|  | Restaurant 5 | Salmon | NS | Atlantic salmon |
| Concepción | Restaurant 1 | Salmon | NS | Atlantic salmon |
|  | Restaurant 2 | Salmon | NS | Atlantic salmon |
|  | Restaurant 3 | Salmon | NS | Atlantic salmon |
|  | Restaurant 4 | Salmon | NS | Atlantic salmon |
|  | Restaurant 5 | Salmon | NS | Atlantic salmon |
| Temuco | Restaurant 1 | Salmon | NS | Atlantic salmon |
|  | Restaurant 2 | Salmon | NS | Rainbow trout |
|  | Restaurant 3 | Salmon | NS | Chinook salmon |
|  | Restaurant 4 | Salmon | Atlantic salmon | Atlantic salmon |
|  | Restaurant 5 | Salmon | NS | Atlantic salmon |
|  | Restaurant 6 | Salmon | NS | Chinook salmon |
|  | Restaurant 7 | Salmon | NS | Atlantic salmon |
|  | Restaurant 8 | Salmon | NS | Rainbow trout |
|  | Restaurant 9 | Salmon | NS | Atlantic salmon |
|  | Restaurant 10 | Salmon | Atlantic salmon | Atlantic salmon |
|  | Restaurant 11 | Salmon | NS | Atlantic salmon |
|  | Restaurant 12 | Salmon | NS | Chinook salmon |
|  | Restaurant 13 | Salmon | NS | Atlantic salmon |
| Valdivia | Restaurant 1 | Salmon | Coho salmon | Atlantic salmon |
|  | Restaurant 2 | Salmon | Atlantic salmon | Atlantic salmon |
|  | Restaurant 3 | Salmon | salmon-trout | Atlantic salmon |
|  | Restaurant 4 | Salmon | NS | Atlantic salmon |
|  | Restaurant 5 | Salmon | NS | Atlantic salmon |
|  | Restaurant 6 | Salmon | Atlantic salmon | Atlantic salmon |
| Osorno | Restaurant 1 | Salmon | Atlantic salmon | Atlantic salmon |
|  | Restaurant 2 | Salmon | Rainbow trout | Rainbow trout |
|  | Restaurant 3 | Salmon | salmon-trout | Rainbow trout |
|  | Restaurant 4 | Salmon | Atlantic salmon | Chinook salmon |
|  | Restaurant 5 | Salmon | Atlantic salmon | Coho salmon |
|  | Restaurant 6 | Salmon | NS | Atlantic salmon |
|  | Restaurant 7 | Salmon | NS | Coho salmon |
|  | Restaurant 8 | Salmon | Atlantic salmon | Atlantic salmon |
|  | Restaurant 9 | Salmon | Atlantic salmon | Atlantic salmon |
|  | Restaurant 10 | Salmon | Atlantic salmon | Atlantic salmon |
| Puerto Montt | Restaurant 1 | Salmon | Atlantic salmon | Coho salmon |
|  | Restaurant 2 | Salmon | Atlantic salmon | Coho salmon |
|  | Restaurant 3 | Salmon | Atlantic salmon | Rainbow trout |
|  | Restaurant 4 | Salmon | NS | Atlantic salmon |
|  | Restaurant 5 | Salmon | Atlantic salmon | Coho salmon |
|  | Restaurant 6 | Salmon | Rainbow trout | Atlantic salmon |
|  | Restaurant 7 | Salmon | Coho salmon | Coho salmon |
|  | Restaurant 8 | Salmon | Coho salmon | Coho salmon |
|  | Restaurant 9 | Salmon | Atlantic salmon | Rainbow trout |
|  | Restaurant 10 | Salmon | NS | Atlantic salmon |
|  | Restaurant 11 | Salmon | Coho salmon | Coho salmon |
|  | Restaurant 12 | Salmon | NS | Rainbow trout |
|  | Restaurant 13 | Salmon | NS | Atlantic salmon |
|  | Restaurant 14 | Salmon | NS | Rainbow trout |
|  | Restaurant 15 | Salmon | NS | Rainbow trout |
| Quellón | Restaurant 1 | Salmon | salmon-trout | Rainbow trout |
|  | Restaurant 2 | Salmon | salmon-trout | Atlantic salmon |

^1^Sushi place refers to restaurants (Japanese or not) and takeaway.

^2^Declared in the menu refers to the name labeled in the sushi menu

3Declared by the salesperson refers to the species declared by salesperson in the sale point

^4^RFLP refers to the species identify using molecular approach.
