# Supplementary material for: Chilean Salmon Sushi: Genetics Reveals Product Mislabeling and a Lack of Reliable Information at the Point of Sale
Source: Foods. 2020 Nov 19;9(11):1699. doi: 10.3390/foods9111699 (PMC7699462; doi:10.3390/foods9111699)
Supplement: Supplementary file 1 [file foods-09-01699-s001.zip › Table S1.docx]

Table S1. List of sequences obtained from GenBank used for support to use of the *Dde*I restriction enzyme as salmonids identifier in this study.

| Species | Sample size | Accession GenBank |
| --- | --- | --- |
| *S*. *salar* | 13 | KF792729, LC012541, NC_001960, U12143.1, JQ390056, KY122205, KY122206, AF165083, FJ435618, FJ435620, EU492280, HQ167697, JX960834 |
|  |  |  |
| *O*. *kisutch* | 20 | EF126369, JF791766, NC_009263, KR422558, AF165079, FJ435609, FJ435610, KM523305, DQ449933, JQ724119, JX185442, JX258853, KJ740756, KJ740757, KJ740758, KJ740759, KJ740760, KJ740761, GU391990, JQ031719 |
|  |  |  |
| *O*. *mykiss* | 20 | AY032629, AY032630, AY032631, AY032632, DQ288268, DQ288269, DQ288270, DQ288271, KP013084, L29771, LC050735, MF621750, MT410879, NC_001717, FJ435597, FJ435598, FJ435599, FJ435600, FJ435601, FJ435602 |
|  |  |  |
| *O*. *tshawytscha* | 18 | AF392054, AJ314566, KF013235, NC_002980, FJ435603, FJ435604, KM523307, KU761862, KU761863, KX958411, DQ449932, HQ167695, JQ724121, JX960819, JX960820, KC795690, KC795691, KU872715 |
