# Supplementary material for: Chilean Salmon Sushi: Genetics Reveals Product Mislabeling and a Lack of Reliable Information at the Point of Sale
Source: Foods. 2020 Nov 19;9(11):1699. doi: 10.3390/foods9111699 (PMC7699462; doi:10.3390/foods9111699)

Supplementary Figure:

Figure S1: Virtual gel for the *in-silico* RFLP analysis of the mitochondrial cytochrome b fragment for sequences obtained from GenBank used for support to use of the *Dde*I.


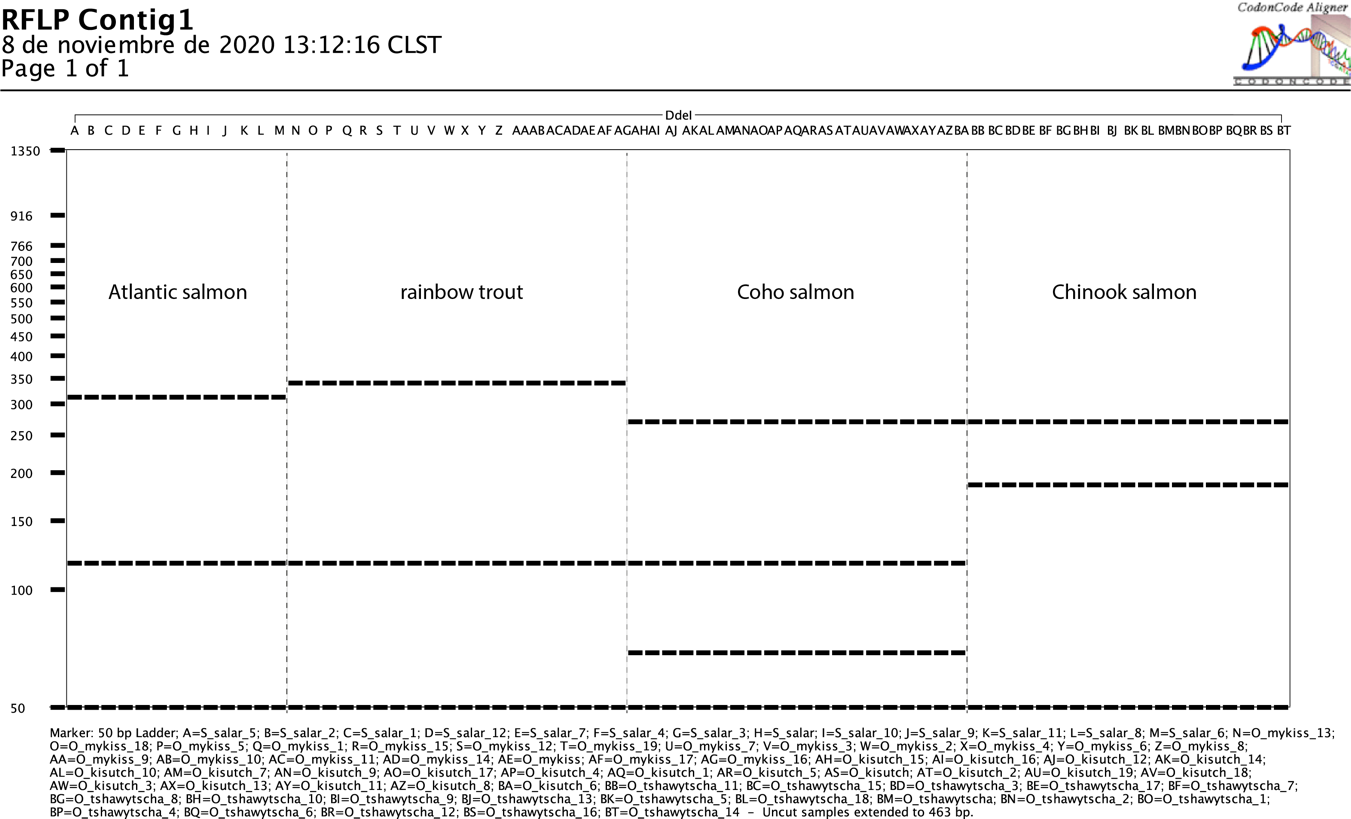

Supplement: Supplementary file 1 [file foods-09-01699-s001.zip › Figure S1.docx]
